# Non-communicable disease risk in female secondary school teachers of Islamabad: a baseline survey from a mixed methods study

**DOI:** 10.3389/fpubh.2026.1858556

**Published:** 2026-08-11

**Authors:** Zoha Imtiaz Malik, Shaheer Ellahi Khan, Abdul Momin Rizwan Ahmad, Saleh A. Alsanie

**Affiliations:** 1 School of Public Health, Health Services Academy, Islamabad, Pakistan; 2 Department of Human Nutrition and Dietetics, NUST School of Health Sciences, National University of Sciences & Technology (NUST), Islamabad, Pakistan; 3 Institute of Health Policy, Management and Evaluation, Dalla Lana School of Public Health, University of Toronto, Toronto, ON, Canada; 4 Department of Health Sciences, University of York, York, United Kingdom; 5 Department of Basic Health Sciences, College of Applied Medical Sciences, Qassim University, Saudi Arabia; 6 Department of Clinical Nutrition, Medical City, Qassim University, Buraydah, Saudi Arabia

**Keywords:** anthropometrics, female teachers, knowledge and awareness, non-communicable diseases, risk factors

## Abstract

**Background:**

Employed females such as school teachers form a particularly vulnerable and overlooked population group, in terms of non-communicable diseases (NCDs), and despite their higher risk, research on female school teachers remains limited.

**Objective:**

The current study aimed to assess NCDs related risk factors, and their knowledge in female secondary school teachers.

**Methods:**

A cross-sectional baseline survey was conducted for a quasi-experimental mixed methods study on 130 female secondary school teachers, selected using two stage sampling, with the first stage involving purposive selection of 08 secondary federal government public schools of Islamabad, and the second stage involving simple random selection of female teachers employed at these schools. Data was collected using pre-validated questionnaires and analyzed using SPSS version 27. Chi-square/Fisher exact test and binary logistic regression were applied to determine associations between variables.

**Results:**

Out of the 130 participants, 63.8% were aged 41–45 years age group, 89.2% had a post-graduate degree, and 90% participants earned more than 150,000 PKR per month. About 38.5% consumed fruits every 3–4 days, 88.5% claimed they did not know how many servings of fruits and vegetables they consumed, and 16.2% participants said they often add salt to their foods before or while eating. About 63.8% participants said they walk for at least 30 min per day, while 31.5% spent more than 6 h while sitting or lying down. The mean BMI was reported to be 27.09 ± 4.94, mean waist circumference (WC) was 95.01 ± 10.05, while mean WtHR was 0.88 ± 0.04. The percentage of participants with moderate level of HTN, CVDs, and DM were 61.5, 76.9, and 64.6%, respectively. Household income (*p* = 0.022), servings of fruits and vegetables (*p* < 0.001), and moderate physical activity (*p* = 0.041) were significantly associated with DM.

**Conclusion:**

This study’s findings reflected high levels of moderate NCDs knowledge, however, participants exhibited increased behavioral and anthropometric NCDs risk factors. This reflects a lack of translational knowledge despite general NCDs awareness in the target population.

## 1 Introduction

Non-communicable diseases are the major contributors to global deaths with 55 million NCDs attributed deaths projected in 2030. The top four leading NCDs include cancer, cardiovascular diseases (CVDs), diabetes, and chronic respiratory diseases. These chronic diseases are also associated with socio-economic and gender related health disparities in various regions of the world. Additionally, health and nutrition awareness, behavior risk factors including unhealthy diets, sedentary lifestyles, and tobacco and alcohol consumption, and access to healthcare services, all contribute significantly to NCDs development (1). Gender is a major social determinant of NCDs and imposes different disease burdens on men and women. Females face greater socio-economic disadvantages coupled with greater household responsibilities, and limited health related autonomy, which renders them susceptible to poor disease prevention and management (2). Females also depict a higher rate of lifestyle and metabolic risk factors clustering. This places them at a higher risk of developing NCDs as compared to men, as evidenced by diabetes prevalence prediction for 2030 (33.5% in women vs. 24% in men). Women in urban areas are more likely to be overweight or obese while in contrast, those in rural areas may be underweight, with both nutritional states leading to metabolic NCDs risk factor development. Gender disparities also exist in accessing healthcare which results in late diagnosis of NCDs in women resulting in poor prognosis and management (3). Similar to the global NCDs trends, South Asian countries also report an increase in their prevalence and annually 9 million deaths are attributed to these diseases (4). Pakistan also reports a 58% NCDs associated deaths, with a 12.5% prevalence of NCD risk factors and 9% prevalence of diabetes in Pakistani women (5).

The increased contribution of women in the workforce has put them at a heightened risk of developing different NCDs risk factors, as they now bear additional household and work related responsibilities. Added stress, increased work burden, hazardous environmental and workplace exposures, all contribute significantly to NCDs associated mortality. Working women exhibit up to 77% obesity, 51% low physical activity, 69% unhealthy diets, and 44% dyslipidemia prevalence (6). In rural working women, occupation stress is coupled with socio-economic challenges such as lack of health and nutrition literacy, poor financial resources, inaccessibility to healthcare facilities, and increased family responsibility due to living in joint families. The aforementioned factors may limit their intake of nutritious foods, participation in healthy behaviors, and exacerbate existing health issues (7). In contrast the working class belonging to upper socio-economic strata, may indulge in sedentary lifestyles, and increased intake of energy dense and highly processed foods, which may then heighten their risk of chronic diseases. The most prevalent lifestyle risk factors among employed adults include decreased fruits and vegetables intakes and increased alcohol consumption (8).

Among working adults, teachers form a unique vulnerable group in terms of NCDs, as they are evidenced to exhibit higher NCDs prevalence as compared to other occupational groups. These statistics can be attributed to their longer working hours, increased workloads, stressful environments, sedentary nature of their job. Remote teaching during the Covid-19 pandemic further exacerbated these risk factors, leading to increased prevalence of diabetes, hypertension, and cardiovascular diseases. Additionally, older age, higher income, being unmarried, years of experience, and negative health perceptions, have all been associated with increased NCDs risk in teachers (9). Furthermore, it is evidenced that teachers’ health significantly impacts their students’ health and nutrition behaviors and habits, as well as the teacher’s own teaching abilities. Unhealthy eating habits are further associated with decreased work productivity, and increased absenteeism. In South Asian countries, teachers’ lifestyle may be influenced by cultural norms, which is depicted in lower physical activity levels and decreased health services use in female teachers (10). Furthermore, teachers reportedly have higher absence rates, with female teachers taking greater number of sick leaves as compared to male teachers. The teaching workforce is predominantly female in majority countries throughout the world, and they present with their unique health challenges requiring tailored interventions (11). Additional studies have associated teaching with various metabolic risk factors, thereby making teachers susceptible to various non-communicable diseases. Evidence has suggested a clustering of cardio-metabolic risk factors, such as increased systolic blood pressure in both male and female teachers. Therefore, interventions focusing on nutrition, physical activity and stress management have improved teachers’ health profiles in several countries (12). Female teachers have also reported a lack of recognition regarding women’s health at their workplaces. Lack of research on women’s health and its comparative complexity as compared to men’s health adds to further health challenges and lack of support and understanding from their work supervisors in case of illness related absences (13).

Despite these facts, school teachers’ health, particularly female teachers, remains underexplored. Evidence on the topic is especially scarce in low and middle income countries (LMICs), such as Pakistan, which currently bears a major NCDs burden. Investigating the prevalence and associated risk factors for these chronic diseases in school teachers is pivotal for strengthening school health promotion initiatives. Moreover, it addresses the often overlooked domain of occupational health within public health, which is a significant, yet under-researched area in LMICs, Even though current literature has assessed NCDs risk factors among school teachers, studies on female teachers and their NCDs risk remain limited, particularly in the Pakistani context. Additionally, very few studies have explored baseline NCDs risk in female teachers as part of and to inform a larger lifestyle intervention study. The current study therefore, targets female teachers of public secondary schools in Islamabad, Pakistan. It aims to assess their behavioral and anthropometric NCDs risk factors, as well as their knowledge and awareness of these diseases and their risk factors. The data generated from this study will then be used to develop workplace specific lifestyle interventions for occupational health promotion and disease prevention.

## 2 Materials and methods

### 2.1 Study design

The study was cross-sectional baseline survey which was conducted as the first phase of a quasi-experimental mixed methods research.

### 2.2 Study area and duration

The study was conducted in 08 secondary federal government public schools of Islamabad Capital Territory (ICT), Pakistan. The data collection for the baseline survey was carried out over a period of 02 months.

### 2.3 Study population

The study population consisted of female teachers permanently employed at these public secondary schools. Female teachers were specifically selected in the study due to their unique socio-cultural, occupational, and household challenges that may increase their risk of NCDs. Additionally, there is a lack of research on female teachers and their risk of NCDs, and owing to the gender specific determinants of these chronic diseases, the study exclusively focused on women to inform gender sensitive lifestyle interventions. Male teachers were excluded from the study, which limits the generalizability of the study’s findings to male teachers and other occupational sectors.

The females were included in the study if they were aged between 30 to 45 years, they were not presently diagnosed with a non-communicable disease, and they had one or more risk factors of NCDs, such as BMI > 25 kg/m^2^, waist circumference > 88 cm, fruit and vegetable intake < 5 servings per day, and physical activity < 150 min/week. The participants were required to have at least one NCDs risk factor as the broader objective for the subsequent quasi-experimental study aimed at assessing the impact of the implemented lifestyle intervention in female teachers with elevated risk of NCDs. Female teachers who were undergoing any medical or herbal treatment for any kind of NCD or other disease, were pregnant or lactating, and those who would not be present in the school within the next 9 months, were excluded from the study.

### 2.4 Sample size

The sample size was calculated, for the subsequent quasi-experimental intervention phase of the study, using the following formula:

$$n=[2\times (\sigma^{2})\times {\left(Z₁-\alpha /₂+Z₁-\beta \right)}^{2}]/d^{2}$$

BMI was the primary outcome for the study, and hence sample size calculation was based on the expected change in BMI from the subsequent intervention. Here the standard deviation was 0.96, which was derived from a similar Indian study which also had BMI has a primary study outcome (14), alpha was 0.05, power of study was kept at 80%, effect size at 0.5, and non-response rate of 10% was considered. The sample size came out to be 130 participants. This same sample size was used for this baseline survey and then for the following intervention implementation.

### 2.5 Sampling technique

The current study used a two-stage sampling technique to select the schools in the 1st stage, and then teachers from each school were selected in the 2nd stage.

In the first stage, all federal government secondary schools were considered clusters, and the list of these schools was considered the sampling frame. As the Pakistan Education Statistics report (2021–22) states that there are 15 teachers on average in each government secondary school, a total of 8 schools were selected to reach a total of 130 teachers. Due to practicality concerns such as school administration permission, cooperation by principals and teachers, and academic commitments, purposive sampling was chosen as the sampling technique.

In the second stage, teachers were selected from each of the 08 schools. The teachers who met the pre-defined inclusion criteria were then subjected to simple random sampling to meet a total sample size of 130.

### 2.6 Data collection tools

Three validated tools were used to collect data on NCDs risk factors, including dietary and physical activity behaviors, and knowledge regarding various NCDs and their risk factors.

#### 2.6.1 WHO STEP wise approach to non-communicable disease risk factor surveillance (STEPS) questionnaire

The first two sections of the STEPS questionnaire, demographic and behavioral risk factor assessment, and anthropometric assessment, were used to collect data on the relevant variables. The third section which focused on the biochemical assessment was not used as the study did not gather data on participants’ biochemical profiles.

#### 2.6.2 General knowledge and attitudes related to non-communicable diseases (GK), NCD behavioral risk factors (RF), and different NCD

This questionnaire was a validated data collection instrument developed by Yenit et al. (15), and is used to assess participant knowledge regarding three major NCDs; diabetes, cardiovascular diseases (CVDs) and hypertension. The questionnaire is divided into three sections for each of the NCDs and knowledge scores of less than 33rd percentile was considered low, 33^rd^ to 66th percentiles were considered moderate knowledge, while scores of more than 66^th^ percentiles were considered high.

#### 2.6.3 National knowledge, attitudes and practices survey on non-communicable diseases

The questionnaire was a validated tool developed by Demaio et al. for the purpose of assessing knowledge and associated attitudes and practices regarding NCDs and their risk factors (16). The questionnaire was adapted according to the Pakistani context, such as by replacing the country’s name in region specific questions (Ethiopia with Pakistan), while the original content, question structure, and scoring remained exactly the same. Knowledge items were scored 1 for correct and 0 for incorrect/do not know. Attitude statements were scored on a Likert scale ranging from 1 for strongly disagree to 4 for strongly agree. Practice item were coded as 1 for healthy practices and 0 for unhealthy behaviors.

### 2.7 Data analysis

Data collected using the above mentioned tools was analyzed using SPSS version 27. Descriptive statistics were applied to determine frequency and percentages for categorical data, while mean and standard deviation was calculated for continuous data. Inferential statistics including chi-square/Fisher exact test and binary logistic regression were applied to determine associations between variables. *p*-value of 0.05 was considered significant while keeping confidence interval at 95%.

### 2.8 Ethical approval

Ethical approval for the study was obtained by the Institutional Review Board (IRB) of the Health Services Academy (HSA), Islamabad, Pakistan, via letter number F. No. 00038/HSA/PhD-2023. Approval was also obtained from the Federal Directorate of Education (FDE), Islamabad, for the conduction of the study via letter numbers (No. F.1-3/2024-(CA)FDE) and (No. F.1-3/2025-(CA)FDE). The study was conducted in accordance with the local legislation and institutional requirements and in accordance with the Declaration of Helsinki. The participants provided their written informed consent to participate in this study.

### 2.9 Published protocol

The protocol for this study has already been published in “Frontiers in Public Health” under section “Public Health Education and Promotion” and can be found at: https://doi.org/10.3389/fpubh.2025.1641499.

## 3 Results

### 3.1 Socio-demographic profile of study participants

Our study included 130 total female participants, as shown in Table 1, out of which 63.8% were in the 41–45 years age group, 89.2% had a post-graduate degree, majority (61.5%) of the participants were Punjabi, 77.7% were married, while 90% participants earned more than 150,000 PKR per month.

**Table 1:** Socio-demographic profiles of the study participants.

Socio-demographic variable	*N* (%)
Age group	
30–35 years	13 (10.0)
36–40 years	34 (26.2)
41–45 years	83 (63.8)
Education level	
Secondary School	1 (0.8)
College/University	13 (10.0)
Postgraduate	116 (89.2)
Ethnicity	
Punjabi	80 (61.5)
Pathan	25 (19.2)
Sindhi	8 (6.2)
Gilgiti	2 (1.5)
Other	15 (11.5)
Marital status	
Never Married	27 (20.8)
Currently Married	101 (77.7)
Widowed	2 (1.5)
Monthly income	
<100,000 PKR	1 (0.8)
100,000–150,000 PKR	12 (9.2)
>150,000 PKR	117 (90.0)

### 3.2 Behavioral measurements (CORE: diet and dietary salt)

Of the total 130 participants, 38.5% consumed fruits every 3–4 days, 88.5% claimed they did not know how many servings of fruits and vegetables they consumed, and 15.4% said they consumed vegetables 1–2 days a week, as shown in Table 2. About 16.2% participants said they often add salt to their foods before or while eating, 30% said they always add salt during cooking, 29.2% often ate foods high in salt, and 15.4% said they consume too much salt.

**Table 2:** Behavioral measurements of participants regarding dietary intakes.

Variable	N (%)
In a typical week, on how many days do you eat fruit?	
Not a single day	2 (1.5)
1–2 days	30 (23.1)
3–4 days	50 (38.5)
Everyday	48 (36.9)
How many servings of fruit do you eat on one of those days?	
Do not know	115 (88.5)
1–2 servings	14 (10.8)
3–4 servings	1 (0.8)
In a typical week, on how many days do you eat vegetables?	
Not a single day	0 (0)
1–2 days	20 (15.4)
3–4 days	61 (46.9)
Everyday	49 (37.7)
How many servings of vegetables do you eat on one of those days?	
1–2 servings	12 (9.2)
3–4 servings	2 (1.5)
5 servings	1 (0.8)
Do not know	115 (88.5)
How often do you add salt or a salty sauce right before or while eating?	
Always	9 (6.9)
Often	21 (16.2)
Sometimes	15 (11.5)
Rarely	19 (14.6)
Never	66 (50.8)
How often is salt or salty seasoning added during cooking in your household?	
Always	8 (6.2)
Often	39 (30.0)
Sometimes	23 (17.7)
Rarely	30 (23.1)
Never	30 (23.1)
How often do you eat processed food high in salt?	
Always	6 (4.6)
Often	38 (29.2)
Sometimes	32 (24.6)
Rarely	23 (17.7)
Never	31 (23.8)
How much salt or salty sauce do you think you consume?	
Too much	20 (15.4)
Just the right amount	74 (56.9)
Too little	33 (25.4)
Far too little	3 (2.3)

### 3.3 Behavioral measurements (CORE: physical activity)

Out of the 130 female school teachers, all reported that their work did not include any rigorous activity (100%), as shown in Table 3. About 63.8% participants said they walk for at least half an hour per day, while only 7 participants reported doing vigorous physical activity that increases breathing rate. Of these 7 participants, 6 were involved in such activities 1–2 days a week and for half an hour.

**Table 3:** Behavioral measurements of participants regarding physical activity.

Variable	*N* (%)
Work involves vigorous-intensity activity (large increase in breathing/heart rate)	
Yes	0 (0)
No	130 (100)
Work involves moderate-intensity activity (small increase in breathing/heart rate such as brisk walking)	
Yes	2 (1.5)
No	128 (98.5)
Walk or use a bicycle to travel to and from places	
Yes	98 (75.4)
No	32 (24.6)
In a typical week, on how many days do you walk or bicycle to get to and from places?	
1–2 days	34 (26.2)
3–4 days	21 (16.2)
>4 days	43 (33.1)
How much time do you spend walking for travel on a typical day?	
Half an hour	83 (63.8)
1–2 h	15 (11.5)
Do any vigorous-intensity sports, fitness or recreational (leisure) activities that cause large increases in breathing or heart rate	
Yes	7 (5.4)
No	123 (94.6)
In a typical week, on how many days do you do vigorous-intensity sports, fitness or recreational (leisure) activities?	
1–2 days	6 (4.6)
3–4 days	0 (0)
>4 days	1 (0.8)
Time spent doing vigorous-intensity sports, fitness or recreational activities on a typical day	
Half an hour	6 (4.6)
1–2 h	1 (0.8)
Do any moderate-intensity sports, fitness or recreational (leisure) activities that cause a small increase in breathing or heart rate	
Yes	2 (1.5)
No	128 (98.5)
In a typical week, on how many days do you do moderate-intensity sports, fitness or recreational (leisure) activities?	
1–2 days	1 (0.8)
3–4 days	0 (0)
>4 days	1 (0.8)
How much time do you spend doing moderate-intensity sports, fitness or recreational (leisure) activities on a typical day?	
Half an hour	0 (0)
1–2 h	2 (1.5)
How much time do you usually spend sitting or reclining on a typical day?	
1–2 h	3 (2.3)
3–4 h	53 (40.8)
5–6 h	33 (25.4)
>6 h	41 (31.5)

### 3.4 Anthropometric measurements of study participants

The mean height and weight of the study participants was 160.40 ± 8.60, and 69.48 ± 12.42, respectively, as shown in Table 4, while the mean BMI was reported to be 27.09 ± 4.94.

**Table 4:** Anthropometric measurements of study participants.

Variable	Mean ± SD (Min–Max)
Height (cm)	160.40 ± 8.60 (125–176)
Weight (kg)	69.48 ± 12.42 (42–110)
Body mass index (kg/m^2^)	27.09 ± 4.94 (16.61–42.24)
Waist circumference (cm)	95.01 ± 10.05 (71–128)
Hip circumference (cm)	107.33 ± 11.32 (80–144)
Waist-to-hip ratio	0.88 ± 0.04 (0.78–0.98)

### 3.5 Knowledge of specific non- communicable diseases

**Figure 1:**
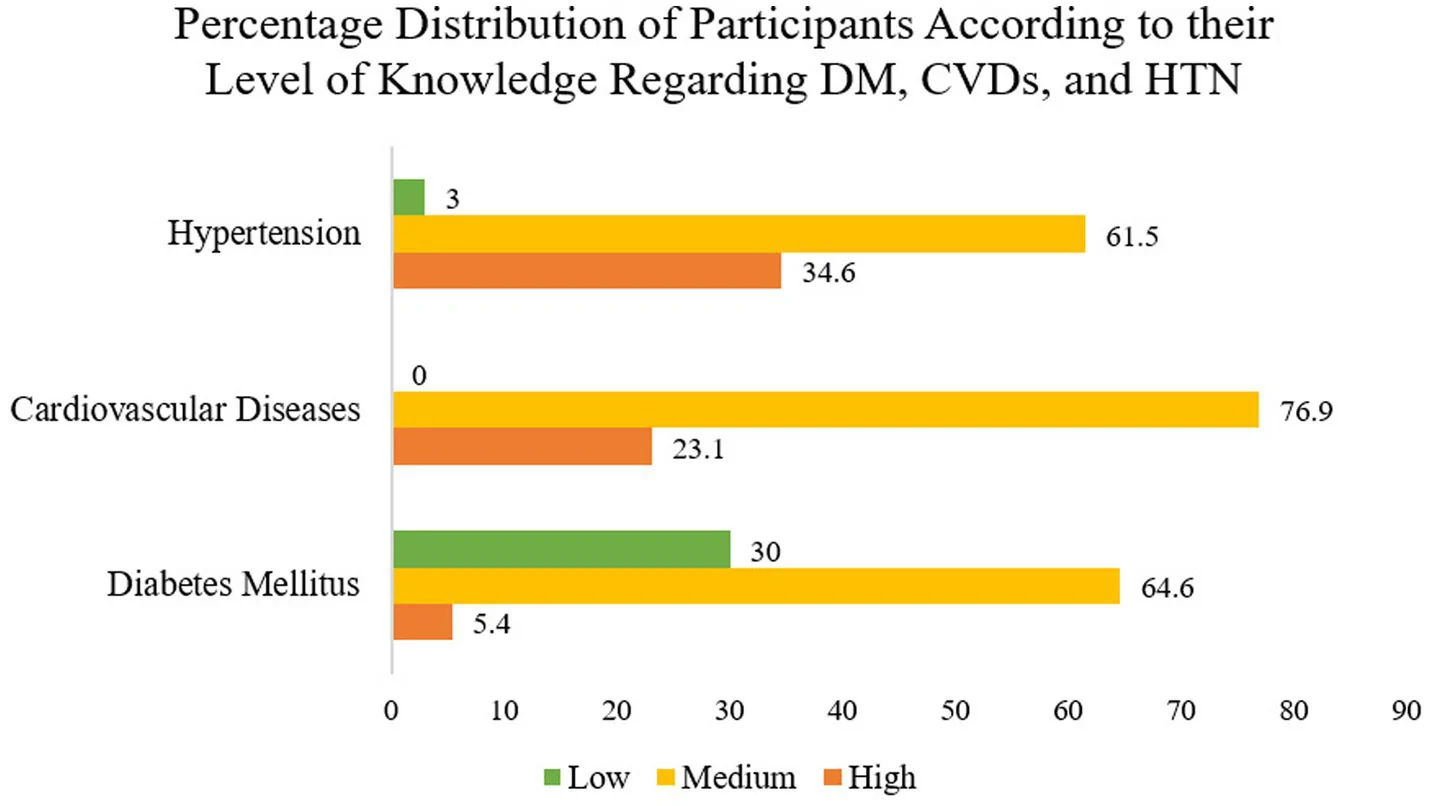
Percentage distribution of participants according to their level of knowledge regarding DM, CVDs, and HTN.

Table 5 summarizes the study participants’ knowledge of prevalent NCDs, including blood pressure, cardiovascular diseases, and diabetes. 76.9% participants said they had heard a little about the term high blood pressure, 86.2% had heard about CVDs, and 76.2% reported they knew a little about diabetes. Furthermore, 66.2% participants were unsure if hypertension was another name for high blood pressure, 56.2% identified diabetes as a cardiovascular diseases, and 20.8% said that diabetes cannot be prevented early. Figure 1 further depicts the percentages of participants falling in low, moderate, and high knowledge levels of each of the three NCDs.

**Table 5:** Participant knowledge of specific non-communicable diseases.

Variable	*N* (%)
How much do you know about high blood pressure?	
Nothing	0 (0)
Only heard the term before	3 (2.3)
A little about it	100 (76.9)
Familiar with it	27 (20.8)
If you have a family history of hypertension, you are at risk of developing high blood pressure	
Yes	115 (88.5)
No	12 (9.2)
Not sure	3 (2.3)
Hypertension is becoming common in Pakistan	
Yes	126 (96.9)
No	3 (2.3)
Not sure	1 (0.8)
Hypertension is another name for high blood pressure	
False	0 (0)
True	44 (33.8)
Not sure	86 (66.2)
Hypertension can be treated with medication	
False	55 (42.3)
True	64 (49.2)
Not sure	11 (8.5)
Lifestyle change such as weight loss can decrease blood pressure	
False	58 (44.6)
True	58 (44.6)
Not sure	14 (10.8)
Lifestyle change such as smoking cessation can decrease blood pressure	
False	7 (5.4)
True	113 (86.9)
Not sure	10 (7.7)
Damage to the kidney can be a sign of high blood pressure	
False	0 (0)
True	104 (80.0)
Not sure	16 (12.3)
Regular exercise reduces blood pressure	
False	5 (3.8)
True	112 (86.2)
Not sure	13 (10.0)
How much do you know about cardiovascular disease?	
Nothing	1 (0.8)
Only heard the term before	9 (6.9)
A little about it	112 (86.2)
Familiar with it	8 (6.2)
The older a person is, the greater his/her risk of having cardiovascular disease	
False	12 (9.2)
True	115 (88.5)
Not sure	3 (2.3)
Keeping blood pressure under control will reduce risk for developing CVD	
False	2 (1.5)
True	126 (96.9)
Not sure	2 (1.5)
Which of the following diseases are cardiovascular (CVDs)?	
Stroke	108 (83.1)
Cancer	9 (6.9)
Diabetes	73 (56.2)
HIV/AIDS	1 (0.8)
High blood pressure	128 (98.5)
Malaria	2 (1.5)
Tuberculosis	2 (1.5)
Which of the following are risk factors for cardiovascular diseases (CVDs)?	
Having regular physical activity	4 (3.1)
High salt intake	72 (55.4)
High blood pressure	97 (74.6)
Fatty meal	119 (91.5)
High sugar intake	43 (33.1)
Avoiding smoking	7 (5.4)
Alcohol consumption	5 (3.8)
Being long-time sitting/sitting idle	43 (33.1)
Which of the following are preventive measures for cardiovascular diseases?	
Regular physical activity	35 (26.9)
Reduce high salt intake	84 (64.6)
Reduce fatty meal	117 (90.0)
Reduce high sugar intake	53 (40.8)
Avoiding smoking	15 (11.5)
Avoid long-time sitting/sitting idle	42 (32.3)
Cardiovascular diseases (CVDs) are becoming common in Pakistan	
False	0 (0)
True	129 (99.2)
Not sure	1 (0.8)
How much do you know about diabetes mellitus?	
Nothing	3 (2.3)
Only heard the term before	9 (6.9)
A little about it	99 (76.2)
Familiar with it	19 (14.6)
Diabetes is when there is too much sugar in the blood	
False	1 (0.8)
True	72 (55.4)
Not sure	57 (43.8)
Type 1 diabetes is a condition of insufficient insulin production	
False	1 (0.8)
True	20 (15.4)
Not sure	109 (83.8)
Type 2 DM is a condition of the body not responding to insulin	
False	1 (0.8)
True	19 (14.6)
Not sure	110 (84.6)
Type 2 DM is common among elders	
False	4 (3.1)
True	28 (21.5)
Not sure	98 (75.4)
Can diabetes be prevented early?	
Yes	78 (60.0)
No	27 (20.8)
Do not know	25 (19.2)
Diabetes can affect vital organs	
False	1 (0.8)
True	124 (95.4)
Not sure	5 (3.8)
Which are signs and symptoms of diabetes mellitus?	
Weight loss	75 (57.7)
Blurred vision	50 (38.5)
Excessive hunger	81 (62.3)
Feeling tired	66 (50.8)
Excessive thirst	86 (66.2)
Frequent urination, often night	82 (63.1)
Very dry skin	8 (6.2)
High blood sugar	7 (5.4)
Slow healing of wounds	16 (12.3)
Complications of DM	
Loss of sensation to feet	114 (87.7)
Damage to the heart	12 (9.2)
Blindness	52 (40.0)
Damage to kidney	41 (31.5)
Damage to brain	9 (6.9)
Diabetes patients should do regular exercise as part of treatment	
Yes	127 (97.7)
No	0 (0)
Not sure	3 (2.3)
How often should diabetes patients do exercise?	
Once a week for at least 30 min	17 (13.1)
All days of the week for at least 30 min	108 (83.1)
Once a month for at least one hour	1 (0.8)
I do not know	4 (3.1)

### 3.6 Association of socio-demographic and behavioral factors with adequate knowledge of HTN, CVD, and DM

Table 6 shows that household income was significantly associated with DM knowledge (*p* = 0.022), and participants earning <150,000 PKR demonstrating adequate DM knowledge as compared to those earning >150,000 PKR. Participants consuming 1–2 servings of fruits and vegetables had adequate DM knowledge as compared to those who did not know how many fruit servings they consumed (*p* < 0.001). HTN and CVDs knowledge were not significantly associated with any socio-demographic or behavioral variable.

**Table 6:** Association of socio-demographic and behavioral factors with adequate knowledge of HTN, CVD, and DM.

Variables	Adequate HTN Knowledge			Adequate CVD Knowledge			Adequate DM Knowledge		
	Yes (*n* = 45)	No (*n* = 85)	*p*-value	Yes (*n* = 30)	No (*n* = 100)	*p-*value	Yes (*n* = 7)	No (*n* = 123)	*p*-value
	n (%)			n (%)			n (%)		
Age group									
30–35 years	7 (15.6)	6 (7.1)	0.319	1 (3.3)	12 (12)	0.285	1 (14.3)	12 (9.8)	0.846
36–40 years	11 (24.4)	23 (27.1)		10 (33.3)	24 (24)		2 (28.6)	32 (26)	
41–45 years	27 (60)	56 (65.9)		19 (63.3)	64 (64)		4 (57.1)	79 (64.2)	
Level of education									
College/University	3 (6.7)	11 (12.9)	0.377	4 (13.3)	10 (10)	0.737	0 (0)	14 (11.4)	1.000
Post graduate degree	42 (93.3)	74 (87.1)		26 (86.7)	90 (90)		7 (100)	109 (88.6)	
Ethnicity									
Punjabi	26 (57.8)	54 (63.5)	0.454	24 (80)	56 (56)	0.274	5 (71.4)	75 (61)	0.407
Pathan	10 (22.2)	15 (17.6)		3 (100)	22 (22)		0 (0)	25 (20.3)	
Sindhi	1 (2.2)	7 (8.2)		1 (3.3)	7 (7)		1 (14.3)	7 (5.7)	
Gilgiti	1 (2.2)	1 (1.2)		0 (0)	2 (2)		0 (0)	2 (1.6)	
Other	7 (15.6)	8 (9.4)		2 (6.7)	13 (13)		1 (14.3)	14 (11.4)	
Marital Status									
Never Married	10 (22.2)	17 (20)	0.766	6 (20)	21 (21)	0.906	3 (42.9)	24 (19.5)	0.156
Ever Married	35 (77.8)	68 (80)		24 (80)	79 (79)		4 (57.1)	99 (80.5)	
Average earnings of the household have been per month									
≤150,000 PKR	7 (15.6)	6 (7.1)	0.137	1 (3.3)	12 (12)	0.297	3 (42.9)	10 (8.1)	0.022*
>150,000 PKR	38 (84.4)	79 (92.9)		29 (96.7)	88 (88)		4 (57.1)	113 (91.9)	
How many servings of fruit do you eat on one of those days?									
Do not Know	39 (86.7)	76 (89.4)	0.504	28 (93.3)	87 (87)	0.630	1 (14.3)	114 (92.7)	<0.001*
1–2	5 (11.1)	9 (10.6)		2 (6.7)	12 (12)		5 (71.4)	9 (7.3)	
3–4	1 (2.2)	0 (0)		0 (0)	1 (1)		1 (14.3)	0 (0)	
How many servings of vegetables do you eat on one of those days?									
1–2	6 (13.3)	6 (7.1)	0.544	2 (6.7)	10 (10)	0.606	6 (85.7)	6 (4.9)	<0.001*
3–4	1 (2.2)	1 (1.2)		1 (3.3)	1 (1)		0 (0)	2 (1.6)	
5	0 (0)	1 (1.2)		0 (0)	1 (1)		0 (0)	1 (0.8)	
Do not Know	38 (84.4)	77 (90.6)		27 (90)	88 (88)		1 (14.3)	114 (92.7)	
How often is salt or salty seasoning added during cooking in your household?									
Always	2 (4.4)	6 (7.1)	0.970	1 (3.3)	7 (7)	0.454	0 (0)	8 (6.5)	0.155
Often	13 (28.9)	26 (30.6)		6 (20)	33 (33)		0 (0)	39 (31.7)	
Sometimes	9 (20)	14 (16.5)		8 (26.7)	15 (15)		1 (14.3)	22 (17.9)	
Rarely	10 (22.2)	20 (23.5)		7 (23.3)	23 (23)		2 (28.6)	28 (22.8)	
Never	11 (24.4)	19 (22.4)		8 (26.7)	22 (22)		4 (57.1)	26 (21.1)	
How much salt or salty sauce do you think you consume?									
Too much	4 (8.9)	16 (18.8)	0.307	4 (13.3)	16 (16)	0.944	1 (14.3)	19 (15.4)	0.208
Just the right amount	27 (60)	47 (55.3)		17 (56.7)	57 (57)		3 (42.9)	71 (57.7)	
Too little	12 (26.7)	21 (24.7)		8 (26.7)	25 (25)		2 (28.6)	31 (25.2)	
Far too little	2 (4.4)	1 (1.2)		1 (3.3)	2 (2)		1 (14.3)	2 (1.6)	
Work involves vigorous-intensity activity (large increase in breathing/heart rate)									
Yes	0 (0)	0 (0)	NA	0 (0)	0 (0)	NA	0 (0)	0 (0)	NA
No	45 (100)	85 (100)		30 (100)	100 (100)		7 (100)	123 (100)	
Work involves moderate-intensity activity (small increase in breathing/heart rate such as brisk walking)									
Yes	1 (2.2)	1 (1.2)	1.000	0 (0)	2 (2)	1.000	1 (14.3)	1 (0.8)	0.105
No	44 (97.8)	84 (98.8)		30 (100)	98 (98)		6 (85.7)	122 (99.2)	
In a typical week, on how many days do you walk or bicycle to get to and from places?									
1–2 days	10 (28.6)	24 (38.1)	0.131	4 (17.4)	30 (40)	0.117	1 (33.3)	33 (34.7)	1.000
3–4 days	5 (14.3)	16 (25.4)		6 (26.1)	15 (20)		0 (0)	21 (22.1)	
>4 days	20 (57.1)	23 (36.5)		13 (56.5)	30 (40)		2 (66.7)	41 (43.2)	
How much time do you usually spend sitting or reclining on a typical day?									
1–2 h	1 (2.2)	2 (2.4)	0.440	0 (0)	3 (3)	0.895	0 (0)	3 (2.4)	0.510
3–4 h	17 (37.8)	36 (42.4)		12 (40)	41 (41)		2 (28.6)	51 (41.5)	
5–6 h	9 (20)	24 (28.2)		7 (23.3)	26 (26)		1 (14.3)	32 (26)	
>6 h	18 (40)	23 (27.1)		11 (36.7)	30 (30)		4 (57.1)	37 (30.1)	

The * represents all statistically significant *p*-values (</=0.05).

### 3.7 Multivariable binary logistic regression of factors associated with adequate HTN, CVD, and DM knowledge

Table 7 shows multivariable binary logistic analysis which found that moderate intensity physical activity at work was significantly associated with adequate DM knowledge (OR = 20.33, 95% CI: 1.13–366.02, *p* = 0.041). Participants ages 30–35 years depicted higher odds of adequate HTN knowledge as compared to those aged 41–45 years (OR = 2.42, 95% CI: 0.74–7.90, *p* = 0.143). Additionally, those with ≤150,000 PKR household income had higher but non-significant odds of adequate HTN knowledge (OR = 2.43, 95% CI: 0.76–7.71, *p* = 0.133). CVDs also showed non-significant associations with all variables, however, those aged 30–35 years (OR = 0.28, 95% CI: 0.03–2.30, *p* = 0.668), and of Pathan ethnicity (OR = 0.32, 95% CI: 0.09–1.17, *p* = 0.084) showed lower odds of adequate CVD knowledge.

**Table 7:** Multivariable binary logistic regression of factors associated with adequate HTN, CVD, and DM knowledge.

Variables	Adequate HTN knowledge		Adequate CVD knowledge		Adequate DM knowledge	
	*p*-value	OR (95% CI)	*p*-value	OR (95% CI)	*p*-value	OR (95% CI)
Age group						
30–35 years	0.143	2.420 (0.741–7.900)	0.236	0.281 (0.034–2.300)	0.668	1.646 (0.169–15.993)
36–40 years	0.985	0.992 (0.423–2.327)	0.459	1.404 (0.572–3.445)	0.813	1.234 (0.215–7.078)
41–45 years		1.000		1.000		1.000
Level of education						
College/University	0.281	0.481 (0.127–1.820)	0.607	1.385 (0.401–4.780)	0.999	NA
Post graduate degree		1.000		1.000		1.000
Ethnicity						
Pathan	0.491	1.385 (0.548–3.498)	0.084	0.318 (0.087–1.165)	0.998	NA
Sindhi	0.267	0.297 (0.035–2.539)	0.316	0.333 (0.039–2.859)	0.513	2.143 (0.219–21.002)
Gilgiti	0.610	2.077 (0.125–34.533)	0.999	0.000 (NA)	0.999	NA
Other	0.295	1.817 (0.595–5.553)	0.199	0.359 (0.075–1.714)	0.951	1.071 (0.116–9.880)
Punjabi		1.000		1.000		1.000
Marital status						
Never Married	0.766	1.143 (0.474–2.758)	0.906	0.940 (0.341–2.597)	0.156	3.094 (0.649–14.751)
Ever Married		1.000		1.000		1.000
Average earnings of the household have been per month						
≤150,000 PKR	0.133	2.425 (0.763–7.714)	0.196	0.253 (0.032–2.030)	0.010	8.475 (1.659–43.283)
>150,000 PKR		1.000		1.000		1.000
How many servings of fruit do you eat on one of those days?						
Do not Know	1.000	NA	1.000	NA	0.999	NA
1–2	1.000	NA	1.000	NA	1.000	NA
3–4		1.000		1.000		1.000
How many servings of vegetables do you eat on one of those days?						
1–2	0.247	2.026 (0.612–6.704)	0.595	0.652 (0.135–3.159)	<0.001	114 (11.77–1104.11)
3–4	0.621	2.026 (0.123–33.286)	0.409	3.259 (0.197–53.874)	1.000	NA
5	1.000	0.00 (NA)	1.000	NA	1.000	NA
Do not Know		1.000		1.000		1.000
How often is salt or salty seasoning added during cooking in your household?						
Always	0.540	0.576 (0.099–3.361)	0.415	0.393 (0.042–3.713)	0.999	NA
Often	0.773	0.864 (0.319–2.341)	0.253	0.500 (0.152–1.640)	0.999	NA
Sometimes	0.855	1.110 (0.363–3.400)	0.524	1.467 (0.451–4.770)	0.291	0.295 (0.031–2.842)
Rarely	0.787	0.864 (0.299–2.498)	0.766	0.837 (0.260–2.699)	0.398	0.464 (0.078–2.751)
Never		1.000		1.000		1.000
How much salt or salty sauce do you think you consume?						
Too much	0.122	0.125 (0.009–1.749)	0.607	0.500 (0.036–6.997)	0.159	0.105 (0.005–2.411)
Just the right amount	0.318	0.287 (0.025–3.318)	0.681	0.596 (0.051–6.987)	0.069	0.085 (0.006–1.213)
Too little	0.327	0.286 (0.023–3.491)	0.729	0.640 (0.051–8.027)	0.151	0.129 (0.008–2.109)
Far too little		1.000		1.000		1.000
Work involves vigorous-intensity activity (large increase in breathing/heart rate)						
Yes	0.731	0.744 (0.139–3.998)	1.000	NA	1.000	NA
No		1.000		1.000		1.000
Work involves moderate-intensity activity (small increase in breathing/heart rate, i.e., brisk walking)						
Yes	0.650	1.909 (0.117–31.260)	0.999	NA	0.041*	20.333 (1.130–366.02)
No		1.000		1.000		1.000
In a typical week, on how many days do you walk or bicycle to get to and from places?						
1–2 days	0.129	0.479 (0.185–1.240)	0.060	0.308 (0.090–1.052)	0.703	0.621 (0.054–7.155)
3–4 days	0.086	0.359 (0.112–1.157)	0.891	0.923 (0.293–2.912)	0.998	NA
>4 days		1.000		1.000		1.000
How much time do you usually spend sitting or reclining on a typical day?						
1–2 h	0.723	0.639 (0.054–7.617)	0.999	NA	0.999	NA
3–4 h	0.241	0.603 (0.259–1.404)	0.640	0.798 (0.311–2.052)	0.256	0.363 (0.063–2.086)
5–6 h	0.143	0.479 (0.179–1.281)	0.576	0.734 (0.249–2.170)	0.278	0.289 (0.031–2.720)
>6 h		1.000		1.000		1.000

The * represents all statistically significant *p*-values (</=0.05).

## 4 Discussion

The current study aimed at assessing female secondary school teachers’ knowledge on the behavioral lifestyle factors, different NCDs, and their risk factors, as well as their anthropometric measures. This is important to evaluate their risk of developing NCDs later in life, and forms a foundation for their prevention. This particular baseline survey was used to inform a subsequent educational lifestyle intervention to promote participant knowledge and awareness of both NCDs and their risk factors. Similar interventions have been implemented globally and have significantly reduced chronic disease risk among the targeted population (17). When assessing the participants’ knowledge about fruits and vegetables consumption, 88.5% were unaware of the servings they were consuming for either food group. Similar finding were reported from a European study which found that despite high knowledge levels regarding the importance of fruits and vegetables, awareness regarding portion sizes and recommended intake remains limited, highlighting a gap between general nutrition literacy and actionable knowledge (18). Another study from Malaysia found that 89.5% of adults did not consume five servings of either fruits or vegetables. Additionally, adequate fruits and vegetables intake was associated with awareness of dietary guidelines, indicating that poor nutrition knowledge is directly linked with lack of recommended intake of fruits and vegetables (19). In terms of salt consumption, 30% participants said they often used salt or some kind of salty seasoning in their cooking, which aligns with regional salt intake patterns, in countries like Pakistan, India, and Bangladesh. Furthermore, the average salt consumption in these countries is nearly double (~10 g/day) than the WHO’s recommendations of 05 g/day. This excessive intake can be attributed to poor perception of high salt intake, habitual practices of sprinkling salt over food, and unawareness of hidden salt sources such as pickles, or spice mixes commonly used while cooking (20). When asked about physical activity behaviors, 75.4% reported walking to and from places, which coincides with the overall high trend of relying on active commuting as part of daily travel. These findings were similar to a Nepalese study which reported that 96% of the participants either walked or cycled as their daily commute, which was considered the biggest contributor to their per day physical activity (21). However, an analysis of the frequency and duration of the aforementioned walking patterns reveals certain limitations. Majority of the participants only engaged in walking for 1–2 days a week, indicating that physical activity is not sustained, hence reducing its overall health benefits. Additionally, 31.5% participants reported sitting for more than 6 h each day, despite engaging in active commute through the day. A study focused on South Asian immigrants residing in Australia reported that 27% had a high sitting time and 34% Pakistanis did not engage in sufficient physical activity, with lack of time being reported as the main barrier (22).

Our study’s participants reported a mean BMI of 27.09 kg/m^2^ demonstrating an overweight profile, along with a mean WC of 95.01 cm, which is again higher than Asia Pacific WC cut off points for females (≥ 80 cm). The Pakistan National Diabetes Survey (NDSP 2016–17) reported the average BMI among Pakistani adults to be 23 kg/m^2^ with women reporting higher adiposity levels as compared to males. Additionally, 30% of the women in the sample were overweight or obese when the cut-off was BMI ≥ 25 (23). A similar study from Pakistan reported mean BMI in employed females to be 32.4 ± 12.4 and that of unemployed females was 30.8 ± 11.8. Furthermore, majority of the study’s sample fell in level 1 obesity. The employed females had higher intakes of processed foods, availability of house help for chores and other facilities, and limited time for physical activity, all of which can be associated with higher BMI values (24). Furthermore, urban Pakistani women tend to report higher BMI and WC values, which are subsequently linked with increased cardio-metabolic risk. Pakistani women also report higher odds of obesity as compared to males, and rural and urban females have 1.80 and 2.62 odds of being obese, respectively, indicating increased obesity risk among urban females (25). A study conducted on Pakistani female teachers reported a 39.0% prevalence of overweight females. Female teachers belonging to high income households had higher likelihood of being overweight, as higher socio-economic status is associated with unhealthy lifestyles. Furthermore, for every unit increase in age, the odds of being overweight increase by 14.8 times. This is due to age related decline in metabolic rate and reduced physical activity levels. Additionally, females with higher levels of education and those teaching in secondary schools are also more likely to have higher BMIs (26). This again can be linked to higher financial status which may result in sedentary lifestyles and unhealthy food choices. It is important to understand these disparities when tailoring lifestyle interventions for this population group. Overall, the elevated mean BMI and WC levels in our study are indicative of higher NCDs risk in the study population, as well as being comparable to previously reported values in literature.

Our study assessed the knowledge levels for three NCDs; HTN, CVDs, and DM, within the study population. The findings indicated that 61.5% of the female teachers had moderate level of HTN knowledge, while only 3.8% had low knowledge levels. These results are comparable to another Pakistani study which found that adequate HTN knowledge was found in only 33.6% non-hypertensive adults and majority (66.4%) of them had poor HTN knowledge (27). This indicates a severe lack of awareness and understanding about the diseases within the general public. On the contrary, however, another Pakistani study found that individuals diagnosed with HTN demonstrated much higher knowledge about the disease. The study carried out in Karachi reported that 2.1, 79.4, and 18.5% possessed low, moderate, and high levels of HTN knowledge, respectively. These results are indicative of the fact that exposure to healthcare systems and professionals may increase individuals’ awareness of their disease (28). Hence, strengthening of and improving access to preventive healthcare via trained professionals is vital to enhance the public’s awareness of various diseases, thereby resulting in early risk reduction. Studies have highlighted that South Asian women often depict lower HTN levels as compared to men due to discrepancies in education levels, socioeconomic factors, and limited exposure to healthcare (29). Due to our study sample comprising of employed females, majority of whom had post-graduate degrees, the findings reported a higher percentage of females demonstrating moderate HTN levels. However, it should be stated that despite a moderate knowledge majority, the sample may still reflect functional inadequacy in terms of HTN awareness. The current study also reported a 76.9% moderate and 23.1% high CVDs knowledge prevalence, with a notable 0% prevalence of low knowledge. These findings are similar to other studies which reported that women depict low to moderate CVDs knowledge. In Korea, 52% women were unaware of CVDs, 19% in the UAE were aware of the associated risks of CVDs, while women in Italy had a low perceived risk of CVDs (30). A Pakistani study assessing CVDs risk awareness in pregnant females found that only 31% had good knowledge of the disease, despite 26% agreeing that it was one of the leading causes of mortality. They also demonstrated poor knowledge of CVDs risk factors and complications, thereby stressing the need for educational programs that inculcate CVDs awareness in the general population as well and vulnerable groups, such as pregnant females (31). Furthermore, studies have shown that women who have post-graduate level of education are more likely to have higher CVDs knowledge as compared to those with other secondary level education (32). This evidence strengthens our study’s high prevalence of moderate level CVDs knowledge, as majority of the participants had post-graduate degrees. Both HTN and CVDs were not significantly associated with any of the socio-demographic variables.

Our study also reported that majority (64.6%) of the participants had moderate DM knowledge, while only 5.4% had high DM knowledge, indicating general awareness of the disease yet lack of comprehensive understanding. A recent study from Pakistan also reported higher rates of moderate level knowledge (48.7%) as compared to high (24.8%), and low (22.9%) DM knowledge among type 2 diabetic patients. The higher prevalence of high knowledge levels can again be associated with increased healthcare exposure after diagnosis (33). Additionally, a scoping review on DM knowledge in Pakistan demonstrated alarmingly high levels of poor knowledge ranging from 69–79%, with high knowledge scores as low as 3–40%. Additionally women demonstrated better DM knowledge as compared to men, however, only 7% prevalence of good DM related practices were observed, indicating that moderate to high level knowledge does not always translate to optimal DM related health actions (34). DM knowledge was significantly associated with household income with those earning less than 150,000 PKR depicting higher awareness of DM knowledge (*p* = 0.022). This finding is contradictory to existing literature which depicts higher income to be significantly associated with increased DM knowledge (*p* = 0.00) as a recent Pakistani study reported that individuals with higher income (>150,000 PKR) had a 45.7% prevalence of adequate DM knowledge which was higher than all other income groups (35). The discrepancy in our result can be attributed to the higher DM burden in lower income groups leading to more exposure to health care systems resulting in increased awareness of the disease. Additionally, DM knowledge was strongly associated with moderate intensity physical activity (OR = 20.33, *p* = 0.041), which indicates that study participants who engaged in healthier lifestyles including physical activity were more aware of DM and its risks. Another study reported that type 2 diabetics had 66.6% good physical activity knowledge which was also significantly associated with improved DM knowledge (36). Lastly, our study also found a significant association between DM knowledge and consuming 1–2 servings of fruits and vegetables, which is further supported by literature as a similar study found that 48% diabetics consumed only 2–3 servings of fruits and vegetables daily and were confused about the recommended number of servings. Furthermore, daily fruits and vegetables intake was only reported by 12 and 16% of the study’s population, again coinciding with our study’s results which also reported low fruit and vegetable intake patterns (37). In another study, teachers pointed out cooking skills as barriers to healthy eating. They mentioned a lack of cooking competence as a barrier to incorporate healthy recipes, including fruits and vegetables, in their daily meals. Additionally, low food budget, time constraints, and lack of cooking staff also hindered the adoption of healthy eating practices (38).

The study has several strengths, as firstly this is among the few studies specifically targeting female secondary school teachers in Pakistan, a vulnerable yet underexplored occupational group. Furthermore, the study provides a comprehensive assessment of socio-demographics, anthropometrics, and lifestyle behaviors, all of which provide important evidence to develop school based health promotion and disease prevention interventions. Additionally, probability sampling of teachers from within selected schools enhanced the study’s validity. The study also has limitations that need to be acknowledged. Firstly, the study population was intentionally selected as female teachers with elevated NCDs risk, which may limit the generalizability of the findings for the general population of female teachers, male teachers, and other occupational groups. Secondly, due to administrative restriction and practicality concerns, schools selected for the study were done via purposive sampling which may limit generalizability of the results. Thirdly, some variables were self-reported and hence, may be subject to recall and desirability bias. Lastly, our study did not include biochemical assessment of the study participants, due to financial and logistical constraints. Hence, the NCDs risk assessment of participants was based on anthropometric and behavioral risk factors, which may not provide a holistic evaluation of their cardio-metabolic risk profile.

The study has several important implications for future research, policy, and practice. As NCDs continue to grow in incidence and prevalence globally, the current study’s findings can be used to inform future longitudinal research on NCDs, particularly in under-explored groups such as female teachers. These studies are necessitated to improve teachers’ health outcomes and promote workplace lifestyle interventions. Additionally, the findings reflect the need for integration of health in the education system, with existing educational policies being refined to incorporate occupational health policies in Pakistan. NCDs related awareness, screening, and counselling sessions with appropriate resources being provided to engage in healthy lifestyles, should be inculcated within schools. These research and policy measures can help inform strategies to provide healthier environments at the workplace, particularly educational institutes, which then contribute towards the well-being, elevated productivity, and improved quality of life among female teachers.

## 5 Conclusion

The current study aimed at evaluating NCDs related risk factors and knowledge levels in an under-explored population; female school teachers. Overall, the study’s findings indicated higher prevalence of moderate knowledge regarding NCDs, however, in terms of lifestyle behaviors, the participants showcased limited healthy behaviors. This indicates that despite moderate levels of knowledge, practical skills and comprehension remains limited. These findings have important implications for our target group, as female teachers often lack the time needed to engage in NCDs preventive behaviors. This coupled with job related stress, and gender-based socio-cultural barriers limit their ability to translate knowledge into practice. Due to their influential position as role models and mentors to the youth, improving their health and nutritional literacy to promote health and prevent disease, has both primary and secondary public health benefits. Such interventions can help them reduce their chronic disease risk and shape the health behaviors of their students and peers. Therefore, the current study highlights the need for a tailored lifestyle intervention targeted towards female secondary teachers to promote NCDs disease risk awareness, skill development, and motivation for sustainable lifestyle changes.

## Data Availability

The raw data supporting the conclusions of this article will be made available by the authors, without undue reservation.
